# COVID-19 Among American Indian and Alaska Native Persons — 23 States, January 31–July 3, 2020

**DOI:** 10.15585/mmwr.mm6934e1

**Published:** 2020-08-28

**Authors:** Sarah M. Hatcher, Christine Agnew-Brune, Mark Anderson, Laura D. Zambrano, Charles E. Rose, Melissa A. Jim, Amy Baugher, Grace S. Liu, Sadhna V. Patel, Mary E. Evans, Talia Pindyck, Christine L. Dubray, Jeanette J. Rainey, Jessica Chen, Claire Sadowski, Kathryn Winglee, Ana Penman-Aguilar, Amruta Dixit, Eudora Claw, Carolyn Parshall, Ellen Provost, Aurimar Ayala, German Gonzalez, Jamie Ritchey, Jonathan Davis, Victoria Warren-Mears, Sujata Joshi, Thomas Weiser, Abigail Echo-Hawk, Adrian Dominguez, Amy Poel, Christy Duke, Imani Ransby, Andria Apostolou, Jeffrey McCollum

**Affiliations:** ^1^CDC COVID-19 Response Team; ^2^Association of Schools and Programs of Public Health, Washington, DC; ^3^Oak Ridge Institute for Science and Education, Oak Ridge, Tennessee; ^4^Albuquerque Area Southwest Tribal Epidemiology Center, Albuquerque, New Mexico; ^5^Alaska Native Tribal Health Consortium’s Alaska Native Epidemiology Center, Anchorage, Alaska; ^6^California Rural Indian Health Board, Inc., California Tribal Epidemiology Center, Roseville, California; ^7^Great Lakes Inter-Tribal Epidemiology Center, Lac du Flambeau, Wisconsin; ^8^Inter Tribal Council of Arizona, Inc., Tribal Epidemiology Center, Phoenix, Arizona; ^9^Northwest Portland Area Indian Health Board, Northwest Tribal Epidemiology Center, Portland, Oregon; ^10^Portland Area Indian Health Service, Portland, Oregon; ^11^Seattle Indian Health Board, Urban Indian Health Institute, Seattle, Washington; ^12^United South and Eastern Tribes, Inc., Tribal Epidemiology Center, Nashville, Tennessee; ^13^Indian Health Service, Rockville, Maryland; ^14^SciMetrika, LLC, McLean, Virginia.

Although non-Hispanic American Indian and Alaska Native (AI/AN) persons account for 0.7% of the U.S. population,* a recent analysis reported that 1.3% of coronavirus disease 2019 (COVID-19) cases reported to CDC with known race and ethnicity were among AI/AN persons (*1*). To assess the impact of COVID-19 among the AI/AN population, reports of laboratory-confirmed COVID-19 cases during January 22^†^–July 3, 2020 were analyzed. The analysis was limited to 23 states^§^ with >70% complete race/ethnicity information and five or more laboratory-confirmed COVID-19 cases among both AI/AN persons (alone or in combination with other races and ethnicities) and non-Hispanic white (white) persons. Among 424,899 COVID-19 cases reported by these states, 340,059 (80%) had complete race/ethnicity information; among these 340,059 cases, 9,072 (2.7%) occurred among AI/AN persons, and 138,960 (40.9%) among white persons. Among 340,059 cases with complete patient race/ethnicity data, the cumulative incidence among AI/AN persons in these 23 states was 594 per 100,000 AI/AN population (95% confidence interval [CI] = 203–1,740), compared with 169 per 100,000 white population (95% CI = 137–209) (rate ratio [RR] = 3.5; 95% CI = 1.2–10.1). AI/AN persons with COVID-19 were younger (median age = 40 years; interquartile range [IQR] = 26–56 years) than were white persons (median age = 51 years; IQR = 32–67 years). More complete case report data and timely, culturally responsive, and evidence-based public health efforts that leverage the strengths of AI/AN communities are needed to decrease COVID-19 transmission and improve patient outcomes.

Individual COVID-19 case reports submitted to CDC using the CDC COVID-19 case report form^¶^ and through the National Notifiable Diseases Surveillance System** during January 22–July 3, 2020 were analyzed. Laboratory-confirmed^††^ and probable^§§^ COVID-19 cases are reported by state and local health jurisdictions based on reports submitted by health care providers and laboratories. Cases with missing report date were excluded. Probable cases (12,081) and cases among persons repatriated to the United States from Wuhan, China (two cases), and the Diamond Princess cruise ship (41 cases) (*2*) were also excluded. Analysis was limited to the 23 states with >70% complete race/ethnicity information and five or more laboratory-confirmed cases each among AI/AN and white persons. Arizona, which accounts for at least one third of all COVID-19 cases among AI/AN persons nationwide, was excluded from analysis because >30% of race/ethnicity data were missing. Because approximately 2.3 million of 5.2 million AI/AN persons identify with multiple races (*3*), AI/AN race/ethnicity was classified as either AI/AN alone or in combination with other races and ethnicities. White (non-Hispanic) was chosen as the comparator group to avoid comparing rates among AI/AN persons to other marginalized populations that experience similar health disparities. Whereas previous reports focused on COVID-19 incidence among black and Hispanic persons, the race/ethnicity categorization in this analysis maximized these data to allow for the calculation of more stable RR estimates. A generalized estimating equations Poisson regression model was used to calculate cumulative incidence (cumulative cases per 100,000 population), RRs, and 95% CIs for AI/AN and white race/ethnicity categories. Generalized estimating equations models, which perform well for estimating rates with correlated data, were used to account for nonindependence (i.e., clustering) by state (*4*). CDC’s National Center for Health Statistics (NCHS) postcensal bridged-race estimates were used as population denominators (*5*). Symptoms, underlying health conditions, hospitalizations, intensive care unit (ICU) admissions, and deaths were not analyzed because a large percentage of these data were missing. Analyses were conducted using SAS software (version 9.4; SAS Institute). 

Among the 1,613,949 laboratory-confirmed COVID-19 cases voluntarily reported to CDC during January 22–July 3, 2020, 424,899 (26.3%) were reported by the 23 included states. Among these cases, 340,059 (80.0%) had complete race/ethnicity data, including 9,072 (2.7%) among AI/AN persons and 138,960 (40.9%) among white persons. These cases represented 51% of 17,709 reported cases among AI/AN persons and 41% of 339,789 reported cases among whites in all U.S. states and territories. Among the 340,059 cases with complete race/ethnicity data, the cumulative incidence among AI/AN persons was 594 cases per 100,000 (95% CI = 203–1,740), 3.5 (95% CI = 1.2–10.1) times that among white persons (169 per 100,000; 95% CI = 137–209). The magnitude of this reported RR estimate is affected by the elevated RR in New Mexico (RR = 14.9).^¶¶^ Median age among AI/AN and white patients was 40 years (IQR = 26–56 years) and 51 years (IQR = 32–67 years), respectively. AI/AN persons with COVID-19 tended to be younger than white persons with COVID-19: a higher proportion of AI/AN patients were aged <18 years (12.9%) and a smaller proportion were aged ≥65 years (12.6%), compared with white patients aged <18 and ≥65 years (4.3% and 28.6%, respectively) (Table).

**TABLE:** Demographic characteristics and data quality among laboratory-confirmed COVID-19 cases, by race/ethnicity — 23 states,* January 31–July 3, 2020

Characteristic	No. (%)	
	American Indian and Alaska Native^†^ (N = 9,072)	White, non-Hispanic (N = 138,960)
**Age group, yrs**		
Median (IQR)	40 (26–56)	51 (32–67)
0–18	1,171 (12.9)	6,000 (4.3)
19–44	4,091 (45.1)	50,772 (36.5)
45–54	1,384 (15.3)	19,923 (14.3)
55–64	1,284 (14.2)	22,518 (16.2)
≥65	1,141 (12.6)	39,737 (28.6)
Missing	1 (—)	10 (—)
**Sex**		
Female	4,819 (53.5)	72,921 (52.6)
Male	4,181 (46.5)	65,701 (47.4)
Missing	72 (—)	338 (—)
**Symptoms known^§^**		
Yes	998 (11.0)	39,225 (28.2)
No	8,074 (89.0)	99,735 (71.8)
**Underlying health conditions known** ^¶^		
Yes	762 (8.4)	37,993 (27.3)
No	8,310 (91.6)	100,967 (72.7)
Hospitalization status** **known^††^**		
Yes	2,197 (24.2)	109,638 (78.9)
No	6,875 (75.8)	29,322 (21.1)
**ICU admission status known^††^**		
Yes	855 (9.4)	37,150 (26.7)
No	8,217 (90.6)	101,810 (73.3)
**Death status known^††^**		
Yes	2,039 (22.5)	103,371 (74.4)
No	7,033 (77.5)	35,589 (25.6)

**Abbreviations:** COVID-19 = coronavirus disease 2019; ICU = intensive care unit; IQR = interquartile range.

* Alabama, Alaska, Florida, Iowa, Kansas, Kentucky, Maine, Michigan, Minnesota, Mississippi, Missouri, Montana, Nebraska, Nevada, New Hampshire, New Mexico, North Carolina, Ohio, Oregon, Tennessee, Utah, Wisconsin, and Wyoming.

^†^ Alone or in combination with other races and ethnicities.

^§^ Symptoms were classified as “known” if any of the following symptoms were reported as present or absent: fever (measured >100.4°F [38°C] or subjective), cough, shortness of breath, wheezing, difficulty breathing, chills, rigors, myalgia, rhinorrhea, sore throat, chest pain, nausea or vomiting, abdominal pain, headache, fatigue, diarrhea (≥3 loose stools in a 24-hour period), or other symptom not otherwise specified on the form.

^¶^ Underlying health conditions were classified as “known” if any of the following conditions were reported as present or absent: diabetes mellitus, cardiovascular disease (including hypertension), severe obesity (body mass index ≥40 kg/m^2^), chronic renal disease, chronic liver disease, chronic lung disease, immunocompromising condition, autoimmune condition, neurologic condition (including neurodevelopmental, intellectual, physical, visual, or hearing impairment), psychologic/psychiatric condition, and other underlying medical condition not otherwise specified.

** Includes hospitalization with or without ICU admission.

^††^ Hospitalization, ICU admission, and death status were considered known if the response was “yes” or “no” (not “missing” or “unknown”).

Completeness of data on underlying health conditions (e.g., cardiovascular disease and diabetes), symptoms, hospitalization status, ICU admission, and death was lower for AI/AN patients than for white patients. Data on underlying health conditions were available for 762 (8.4%) AI/AN patients and 37,993 (27.3%) white patients, and symptom data were available for 998 (11.0%) AI/AN patients and 39,225 (28.2%) white patients. Whereas hospitalization status, ICU admission status, and vital status (i.e., outcome of death) were known for 78.9%, 26.7%, and 74.4%, respectively, of white COVID-19 patients, this information was available for approximately one third of those percentages of AI/AN patients (24.2%, 9.4%, and 22.5%, respectively). Because of the high prevalence of these missing data elements among AI/AN patients, analysis to identify overall prevalence, possible risk factors for COVID-19, and patient outcomes was not possible.

## Discussion

In 23 states with sufficient COVID-19 patient race/ethnicity data, the overall COVID-19 incidence among AI/AN persons was 3.5 times that among white persons. Although this disparity is mostly influenced by the elevated RR in New Mexico, variability in the RR among states is reflected in the wide confidence interval (95% CI = 1.2, 10.1). Among 345,093 COVID-19 cases meeting the study inclusion criteria, 2.7% of cases occurred in AI/AN persons, more than twice the percentage of non-Hispanic AI/AN cases reported in CDC COVID-19 case surveillance data from all states (1.3%) (*1*). However, this analysis included AI/AN persons who identified as multiple races and ethnicities, which increased AI/AN case identification by 4%, from 8,691 to 9,072 cases in the 23 states. The higher proportion of AI/AN persons in this analysis is also the result of the more completely reported race/ethnicity data in these states.

Historical trauma and persisting racial inequity have contributed to disparities in health and socioeconomic factors between AI/AN and white populations that have adversely affected AI/AN communities; these factors likely contribute to the observed elevated incidence of COVID-19 among the AI/AN population (*6*). The elevated incidence within this group might also reflect differences in reliance on shared transportation, limited access to running water, household size, and other factors that might facilitate COVID-19 community transmission (*6*). Although the elevated prevalence of underlying health conditions among AI/AN persons is well documented (*7*,*8*), in this analysis, data on underlying health conditions were unknown or missing for 91.6% of AI/AN patients compared with 72.7% of white patients, preventing examination of the association between underlying health conditions and COVID-19 incidence. The excessive absence of data among AI/AN persons represents an important gap in public health data for AI/AN persons and suggests a need for additional resources to support case investigation and reporting infrastructure in AI/AN communities.

The findings in this report are subject to at least three limitations. First, data are presented as reported to CDC through a passive case surveillance system. Case data are voluntarily reported to CDC by states without active case finding. The high prevalence of missing data on symptoms, underlying health conditions, hospitalization, ICU admission, and death precluded the analysis of these characteristics and outcomes. Missing data likely reflect state, local, and tribal health jurisdictions’ ability to collect these data given their current case loads, incomplete reporting to CDC, or both. Second, this analysis represents an underestimate of the actual COVID-19 incidence among AI/AN persons for several reasons. Reporting of detailed case data to CDC by states is known to be incomplete; therefore, this analysis was restricted to 23 states with more complete reporting of race and ethnicity. As a result, the analysis included only one half of reported laboratory-confirmed COVID-19 cases among AI/AN persons nationwide, and the examined states represent approximately one third of the national AI/AN population.*** In addition, AI/AN persons are commonly misclassified as non-AI/AN races and ethnicities in epidemiologic and administrative data sets, leading to an underestimation of AI/AN morbidity and mortality (*9*). Finally, the NCHS bridged-race estimates used as population denominators are known to inflate the Hispanic AI/AN population in the United States, resulting in the underestimation of mortality rates among AI/AN populations that include Hispanic AI/AN persons (*10*).

Despite these limitations, these findings suggest that the AI/AN population in the 23 examined states, particularly AI/AN persons aged <65 years, has been disproportionately affected by the COVID-19 pandemic, compared with the white population. More complete case information is needed to more effectively guide the public health response to COVID-19 among the AI/AN population. The collection of this information can be facilitated by more consistent, complete, and accurate collection and reporting by providers, reporting laboratories, and local, state, federal, and tribal public health practitioners, and ensuring the resources to do so. Race/ethnicity data should be collected following best practices for AI/AN data collection, including allowing for the reporting of multiple races and ethnicities and providing adequate training about asking about race and ethnicity in a culturally sensitive manner.^§§§^ Further, among federally recognized tribes, AI/AN race is a political status that confers access to health care services under treaty obligations of the U.S. government^¶¶¶^; these findings highlight the important contribution of adequate health care and public health infrastructure resources to culturally responsive public health efforts intended to sustain the strengths of AI/AN communities.

**Summary**

**What is already known about this topic?**

American Indian and Alaska Native (AI/AN) persons appear to be disproportionately affected by the COVID-19 pandemic; however, limited data are available to quantify the disparity in COVID-19 incidence, severity, and outcomes among AI/AN persons compared with those among other racial/ethnic groups.

**What is added by this report?**

In 23 states with adequate race/ethnicity data, the cumulative incidence of laboratory-confirmed COVID-19 among AI/AN persons was 3.5 times that among non-Hispanic white persons. A large percentage of missing data precluded analysis of some characteristics and outcomes.

**What are the implications for public health practice?**

Adequate health care and public health infrastructure resources are needed to support a culturally responsive public health effort that sustains the strengths of AI/AN communities. These resources would facilitate the collection and reporting of more complete case report data to support evidence-based public health efforts.

## Footnotes

* Based on 2018 U.S. Census single-race estimates for non-Hispanic AI/AN (https://wonder.cdc.gov/Single-Race-v2018.HTML). This represents a subset of the AI/AN population. The total AI/AN population (AI/AN alone or in combination with other races/ethnicities) constitutes 1.4% of the United States population (https://wonder.cdc.gov/Bridged-Race-v2019.HTML). Some have estimated the AI/AN population to constitute up to 1.7% of the United States population (https://www.census.gov/history/pdf/c2010br-10.pdf).

† The first laboratory-confirmed case in the 23 analyzed states was reported on January 31, 2020.

§ Alabama, Alaska, Florida, Iowa, Kansas, Kentucky, Maine, Michigan, Minnesota, Mississippi, Missouri, Montana, Nebraska, Nevada, New Hampshire, New Mexico, North Carolina, Ohio, Oregon, Tennessee, Utah, Wisconsin, and Wyoming.

¶ https://www.cdc.gov/coronavirus/2019-ncov/php/reporting-pui.html.

** https://wwwn.cdc.gov/nndss; https://wwwn.cdc.gov/nndss/covid-19-response.html.

†† A laboratory-confirmed COVID-19 case was defined as a person with a positive test result for SARS-CoV-2, the virus that causes COVID-19, from a respiratory specimen, using real time reverse transcription–polymerase chain reaction testing.

§§ According to the Council of State and Territorial Epidemiologists position statement Interim 20-ID-01, a probable case must 1) meet clinical criteria and epidemiologic criteria with no confirmatory laboratory testing performed; 2) have presumptive laboratory evidence, including detection of specific antigen or antibody in a clinical specimen, and meet clinical criteria or epidemiologic criteria; or 3) meet vital records criteria with no confirmatory laboratory testing performed. (https://cdn.ymaws.com/www.cste.org/resource/resmgr/2020ps/interim-20-id-01_covid-19.pdf).

¶¶ New Mexico accounts for 6,130 (68%) of the AI/AN cases but 16% of the total AI/AN population of the 23 states analyzed.

*** https://wonder.cdc.gov/Bridged-Race-v2018.html.

§§§ https://www.uihi.org/resources/best-practices-for-american-indian-and-alaska-native-data-collection/.

¶¶¶ https://www.ihs.gov/aboutihs/.
